# Bergapten as a Multifunctional Phytochemical in Cancer Therapy: Mechanistic Insights, Pharmacokinetics, and Possible Nanotechnology-Enabled Formulation Strategies

**DOI:** 10.3390/pharmaceutics18081007

**Published:** 2026-08-14

**Authors:** Victória Dogani Rodrigues, Maria Angélica Miglino, Claúdia Rucco Penteado Detregiachi, Sandra Maria Barbalho, Lucas Fornari Laurindo

**Affiliations:** 1 Department of Biochemistry and Pharmacology, School of Medicine, Faculdade de Medicina de Marília (FAMEMA), Marília 17519-030, SP, Brazil; vic8dr@gmail.com; 2 Graduate Program in Structural and Functional Interactions in Rehabilitation, School of Medicine, Universidade de Marília (UNIMAR), Marília 17525-902, SP, Brazil; miglino@unimar.br (M.A.M.); claurucco@unimar.br (C.R.P.D.); smbarbalho@gmail.com (S.M.B.); 3 Division of Cellular Growth, Hemodynamics, and Homeostasis Disorders, Graduate Program in Medical Sciences, Faculdade de Medicina, Universidade de São Paulo (USP), São Paulo 01246-903, SP, Brazil

**Keywords:** bergapten, 5-methoxypsoralen, furanocoumarins, cancer, carcinogenesis, tumor progression, anticancer activity, apoptosis, cell cycle arrest, molecular mechanisms, chemoprevention, nanotechnology, drug delivery systems, pharmacokinetics, translational oncology

## Abstract

Bergapten is a plant-derived linear furanocoumarin widely distributed in Rutaceae and Apiaceae species and increasingly recognized for its multifunctional anticancer potential. This review critically synthesizes current evidence on bergapten’s biosynthesis, physicochemical and pharmacokinetic properties, anti-inflammatory and antioxidant pharmacodynamics, and mechanistic antitumor activity, highlighting its translational relevance within pharmaceutical development. Preclinical studies across diverse malignancies demonstrate pleiotropic anticancer effects. Mechanistically, bergapten activates mitochondrial apoptosis via Bax/Bcl-2 modulation and caspase cascades, induces cell cycle arrest through p53–p21 signaling, and suppresses PI3K/Akt/mTOR and NF-κB pathways. Additional effects include PTEN-mediated autophagy induction, interference with metabolic reprogramming, reversal of multidrug resistance through ABC transporter modulation, and context-dependent photoactivated cytotoxicity. Beyond direct tumor cell targeting, bergapten attenuates pro-inflammatory mediators and regulates redox homeostasis through downregulation of NOX4-derived ROS and activation of Nrf2-driven antioxidant defenses, addressing the redox–inflammatory axis implicated in carcinogenesis. Despite promising mechanistic depth, clinical translation is limited by incomplete human pharmacokinetic data and poor aqueous solubility. Nanotechnology-enabled delivery systems and structural derivatives offer strategies to enhance bioavailability and therapeutic index. Overall, bergapten emerges as a systems-level phytochemical candidate warranting further translational investigation in oncology.

## 1. Introduction

Cancer remains one of the leading causes of morbidity and mortality worldwide, driven by complex genetic, epigenetic, and environmental factors that underlie uncontrolled cell proliferation, resistance to apoptosis, and metastatic progression [1,2]. Despite significant advances in conventional therapies, including surgery, radiation, and chemotherapy, treatment efficacy is often limited by toxicity, resistance, and disease relapse, necessitating the discovery of novel agents with improved safety and multi-targeted anticancer potential [3,4].

In recent decades, phytochemicals—bioactive compounds derived from plants—have garnered considerable interest for their chemopreventive and therapeutic properties across diverse cancer types. These natural products often exert multifaceted mechanisms, including modulation of apoptosis, cell cycle regulation, oxidative stress, and key signaling cascades implicated in carcinogenesis, thereby offering promising adjuncts or alternatives to conventional treatments [5,6,7].

**Figure 1:**
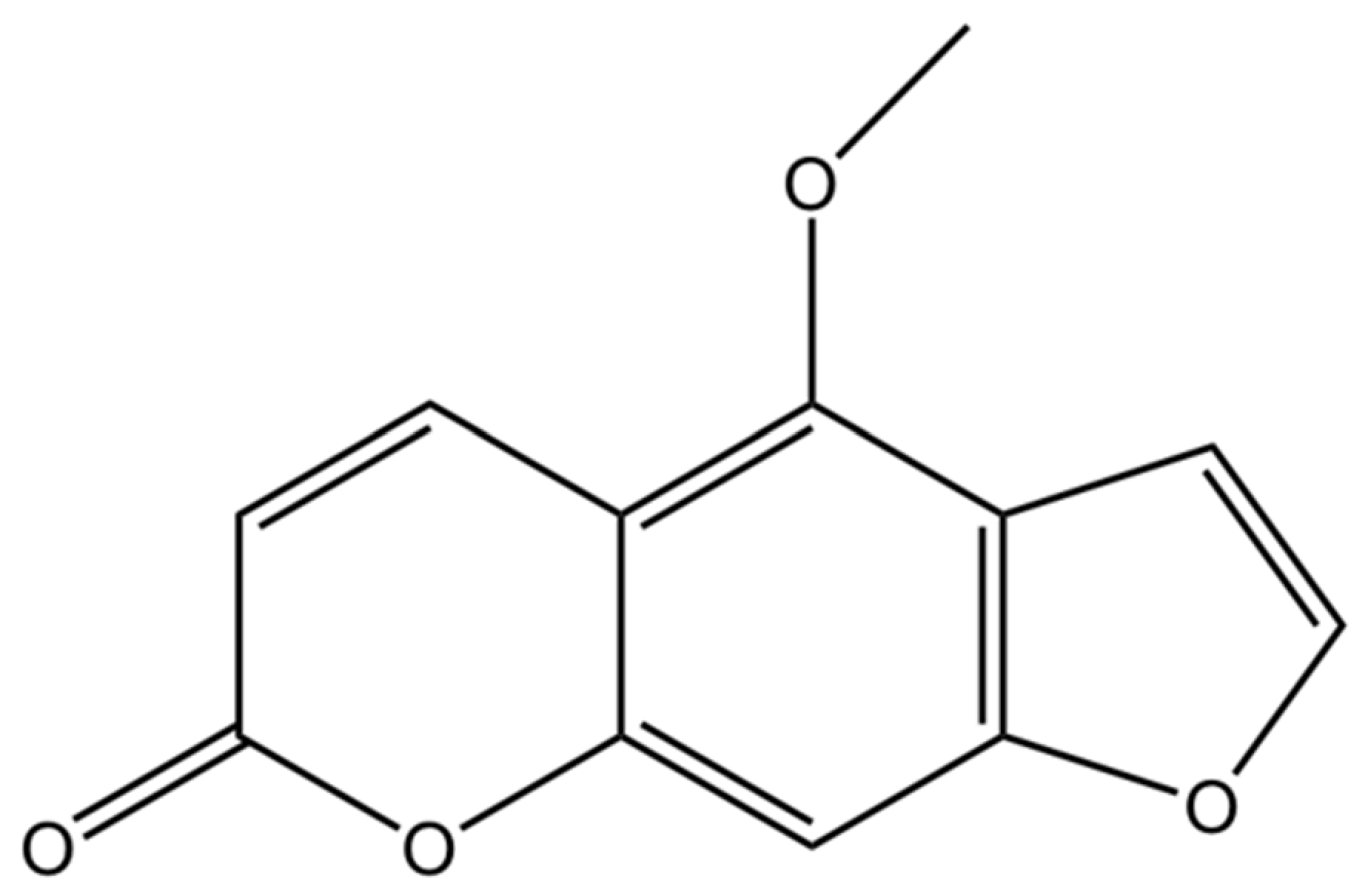
Chemical structure of bergapten (5-MOP), a naturally occurring furocoumarin compound.

Among these, bergapten (5-MOP), a naturally occurring furanocoumarin found in citrus fruits (e.g., lemon and grapefruit) and other botanical sources (e.g., Cnidii Fructus and Fig), has emerged as a compound of notable pharmacological versatility, displaying anticancer, anti-inflammatory, antimicrobial, and organ-protective effects. Although historically recognized for its role in photochemotherapy, particularly when photoactivated by ultraviolet light, accumulating evidence highlights that bergapten exhibits significant anticancer activities independent of photoactivation [8,9]. Figure 1 shows the chemical structure of bergapten.

Initial in vitro investigations demonstrated that bergapten can inhibit cancer cell viability and induce apoptosis in breast cancer cells, in part through the activation of p53 and downstream effectors such as p21, while concurrently impairing survival pathways like PI3K/Akt [9]. Follow-up studies further revealed that bergapten promotes autophagic processes via up-regulation of tumor suppressor PTEN and modulation of autophagy-related proteins (Beclin-1, LC3), suggesting a broader spectrum of antitumor mechanisms beyond apoptosis [10].

Beyond breast cancer, non-UV-activated bergapten has demonstrated antiproliferative and anti-migratory effects across multiple tumor models, including colorectal adenocarcinoma, osteosarcoma, and multiple myeloma, where it induced cell cycle arrest, mitochondrial dysfunction, and caspase-mediated apoptosis, with limited toxicity in normal cell lines [11]. Moreover, mechanistic studies in non-small cell lung cancer have reported bergapten-induced cell cycle arrest and modulation of p53-associated pathways, underscoring its potential relevance in diverse oncologic contexts [12].

The anticancer effects of bergapten also extend to in vivo models, where it exhibited chemopreventive activity against chemically induced oral squamous carcinogenesis by reducing tumor incidence and modulating inflammatory and apoptotic signaling cascades such as PI3K/Akt/mTOR [13]. Additional medicinal chemistry efforts have generated bergapten derivatives with enhanced potency against pancreatic cancer cells, capable of suppressing tumor cell survival and invasion in both organoid systems and zebrafish models [14].

Despite these promising preclinical data, the integration of bergapten into oncology faces several challenges. Pharmacokinetic properties, potential photo-induced toxicity, and the translation of in vitro efficacy to clinical contexts require comprehensive evaluation [8]. Furthermore, the molecular targets and signaling networks modulated by bergapten—such as PI3K/Akt, p38MAPK/NF-Y, and apoptotic cascades—highlight the complexity of its bioactivity and underscore the need for systematic mechanistic dissection [10].

In this comprehensive and critical review, we synthesize current evidence on bergapten’s anticancer mechanisms, assess its therapeutic potential across cancer types, and identify gaps and future directions to inform both basic research and translational strategies.

## 2. Literature Search Strategy

A comprehensive and systematic literature search was conducted to identify relevant studies examining biosynthesis, physicochemical properties, pharmacokinetics, anti-inflammatory and antioxidant pharmacodynamics, and anticancer mechanisms of bergapten. The search was performed in the electronic databases PubMed/MEDLINE, Scopus, Web of Science Core Collection, and Embase from database inception through March 2026. Additional records were identified through manual screening of reference lists of eligible articles and relevant review papers.

Search terms were developed using combinations of MeSH and free-text keywords related to bergapten and its biological activities. The core search string included: “bergapten” OR “5-methoxypsoralen” OR “5-MOP” combined with terms such as “cancer,” “neoplasm,” “carcinoma,” “tumor,” “oncology,” “apoptosis,” “cell cycle,” “PI3K/Akt,” “mTOR,” “NF-κB,” “autophagy,” “oxidative stress,” “reactive oxygen species,” “Nrf2,” “inflammation,” “pharmacokinetics,” “ADME,” “bioavailability,” “metabolism,” “biosynthesis,” “furanocoumarin,” and “nanoparticle” or “nanocarrier.” Boolean operators (AND/OR) were applied appropriately, and database-specific filters were used where necessary.

Studies were included if they were original research articles published in peer-reviewed journals and investigated bergapten in the context of cancer biology, inflammation, oxidative stress, biosynthesis, physicochemical characterization, pharmacokinetics, formulation strategies, or related mechanistic pathways. Both in vitro and in vivo preclinical studies were eligible. Where relevant, mechanistic studies on bergapten derivatives or bergapten-containing formulations were also included. Only articles published in English were considered. Review articles were screened to identify additional primary studies but were not included as primary evidence unless necessary for contextual background. Importantly, no clinical trials evaluating bergapten as an anticancer agent were identified in the searched databases at the time of the final search, and the available evidence is exclusively derived from preclinical in vitro and in vivo studies.

Exclusion criteria comprised conference abstracts without full text, non-peer-reviewed sources, duplicate publications, studies lacking clear methodological description, and reports not specifically evaluating bergapten or clearly distinguishing it from other psoralen derivatives. All retrieved records were screened independently by the authors based on title and abstract, followed by full-text assessment for eligibility. Discrepancies were resolved through discussion and consensus. Data were extracted regarding experimental models, dosing regimens, mechanistic findings, molecular targets, and key outcomes relevant to anticancer, anti-inflammatory, antioxidant, pharmacokinetic, and formulation-related effects.

## 3. Bergapten: From Biosynthetic Pathways to Physicochemical Properties and Pharmacokinetic Profiles

Bergapten is a natural linear furanocoumarin widely distributed in several plant families, particularly the Rutaceae and Apiaceae [15,16,17]. It is significantly isolated from bergamot (*Citrus bergamia*) essential oil [18]. In citrus juices such as bergamot juice, bergapten and related furanocoumarins have been quantified at milligram per liter levels, with bergapten identified alongside bergamottin as one of the major furanocoumarins [19]. Other Rutaceae species, including bitter orange (*Citrus aurantium*) and certain lime varieties, also accumulate bergapten [20,21]. Beyond citrus, bergapten occurs in herbaceous Apiaceae plants such as wild parsnip (*Pastinaca sativa*), and other medicinal or ethnobotanically used seeds and fruits, where it can constitute a substantial proportion of total furanocoumarins [22,23].

Quantitative content of bergapten in these botanical sources is influenced by genetic, environmental, and developmental factors [22,23,24]. To isolate bergapten from complex plant matrices for analytical or pharmacological studies, researchers have applied a range of extraction and purification techniques. Traditional solid–liquid extraction using organic solvents (e.g., methanol, ethanol, or ethyl acetate) remains foundational, often preceded by maceration or Soxhlet extraction to solubilize furanocoumarins from dried plant materials [25,26]. Following crude extraction, chromatographic methods such as column chromatography, HPLC, and SPE are routinely utilized to separate and quantify bergapten and related methoxyfuranocoumarins [26,27]. More sophisticated preparative techniques, including HSCCC and CPC, have been developed to yield high-purity bergapten suitable for bioactivity assays, enabling efficient fractionation without extensive pre-purification steps [28,29]. Emerging advanced methods such as MAE and supercritical fluid extraction have also been explored to enhance extraction efficiency, reduce solvent usage, and improve environmental sustainability of furanocoumarin isolation [25]. Collectively, these botanical sources and extraction strategies underpin the supply of bergapten for research into its bioactivities—including anticancer mechanisms—while also highlighting the variability in content and methodological considerations in natural product research.

In *Ficus carica*, quantitative analysis revealed that bergapten levels varied substantially among eight Algerian fig varieties, ranging roughly from ~114 to ~718.6 µg/g dry weight, with generally higher levels in wood than in bark, and variation across genotypes and fruit color types [30]. Leaf surface analyses of several Rutaceae and Umbelliferous plants—including *Ruta graveolens* and related taxa—showed detectable bergapten alongside psoralen and xanthotoxin at concentrations spanning orders of magnitude on fresh leaves, reflecting species-specific phototoxic profiles [31]. In ribbed celery (*Apium graveolens*), bergapten occurs in trace amounts on leaf surfaces but represents a substantial proportion (up to ~45–71% of total furanocoumarin content) in plant tissues depending on cultivar and plant part [32]. In *Ficus hirta*, different morphological forms showed significant variation in bergapten content in roots correlated with leaf shape, indicating intraspecific chemical diversity [33]. Seeds of *Zanthoxylum schinifolium* have also been quantified to contain bergapten in the range of ~1.7–2.85 mg/g seed, demonstrating that substantial bergapten levels occur across diverse plant species [34].

Overall, bergapten is a widely distributed furanocoumarin whose occurrence spans diverse plant families, notably Rutaceae and Apiaceae. Its content is highly variable, influenced by species, cultivar, plant part, developmental stage, and environmental conditions, with concentrations ranging from trace levels on leaf surfaces to milligrams per gram in seeds. Citrus species, particularly bergamot, remain the richest and most studied sources, while Apiaceae and other medicinal plants contribute substantially to the natural bergapten pool. Isolation of bergapten from these complex matrices relies on a spectrum of methodologies—from classical solvent-based extraction and chromatographic separation to advanced techniques such as HSCCC, CPC, MAE, and supercritical fluid extraction—enabling the preparation of high-purity compounds for analytical and pharmacological studies. Collectively, these findings underscore both the chemical diversity and the methodological considerations inherent in bergapten research, providing a foundation for exploring its biological activities and potential applications.

### 3.1. Biosynthesis of Bergapten: Pathways and Regulatory Mechanisms

Bergapten is formed via specialized secondary metabolism in higher plants, especially within the Apiaceae and Rutaceae families. Its biosynthesis derives from the general phenylpropanoid pathway, a central metabolic route responsible for synthesizing a broad spectrum of natural products, including flavonoids, anthocyanins, tannins, lignans, and coumarins [16,35].

#### 3.1.1. Core Biosynthetic Pathway

The biosynthesis of bergapten begins with phenylalanine, which is deaminated by PAL to produce trans-cinnamic acid. Cinnamic acid is then hydroxylated by C4H to generate p-coumaric acid, a pivotal intermediate entering the phenylpropanoid pathway. Subsequent thioesterification by 4CL yields p-coumaroyl-CoA, the substrate that branches toward coumarin and furanocoumarin formation. From p-coumaroyl-CoA, an ortho-hydroxylation step catalyzed by C2′H is required to form 2,4-dihydroxycinnamoyl-CoA, which undergoes spontaneous lactonization, yielding umbelliferone (7-hydroxycoumarin), the coumarin core serving as the key precursor for furanocoumarins [35].

Umbelliferone then undergoes prenylation with DMAPP to generate demethylsuberosin, a step thought to be catalyzed by a UbiA-type aromatic prenyltransferase (specific enzymes in many species remain to be fully characterized). Demethylsuberosin is further converted through a series of monooxygenase and cyclization reactions to form the furan ring characteristic of psoralen scaffolds. A critical P450-dependent monooxygenase, identified as psoralen synthase in Apiaceae species, catalyzes the oxidative cyclization to yield the parent furanocoumarin psoralen [36,37].

#### 3.1.2. Conversion to Bergapten

The methylation of psoralens represents the final step in bergapten biosynthesis. In particular, BMT catalyzes the transfer of a methyl group from SAM to bergaptol (5-hydroxypsoralen), producing 5-MOP (bergapten). Homologs of BMT have been cloned and functionally characterized in furanocoumarin-producing plants such as *Ammi majus*, and display high substrate specificity for bergaptol over other phenolic compounds [36,37].

Alternative plant systems, such as *Glehnia littoralis*, also exhibit functional BMT activity, suggesting that this O-methylation step is a conserved and essential feature of bergapten biosynthesis across diverse species [38].

#### 3.1.3. Regulatory Mechanisms and Environmental Modulation

Bergapten accumulation in planta is not constitutive; instead, it is dynamically regulated by environmental cues, biotic elicitation, and developmental context. For example, in cell cultures of *Ammi majus*, BMT expression and activity are upregulated in response to fungal elicitors, indicating that defense-related signaling can enhance furanocoumarin biosynthesis [36].

At the broader pathway level, the expression of upstream phenylpropanoid and coumarin biosynthetic genes (e.g., PAL, C4H, C2′H) responds to light, pathogen attack, and abiotic stresses, integrating endogenous and exogenous signals to modulate the pool of intermediates available for furanocoumarin formation. This multi-tiered regulation underscores the role of furanocoumarins as adaptive secondary metabolites [39].

Collectively, bergapten biosynthesis proceeds through the well-established phenylpropanoid pathway, diverging at the 7-hydroxycoumarin stage and progressing via prenylation, oxidative cyclization, and specific methylation steps. Enzyme families central to this pathway include P450 monooxygenases (e.g., psoralen synthase) and SAM-dependent O-methyltransferases (e.g., BMT). The pathway’s regulation integrates developmental programs and stress responses, reflecting the ecological and pharmacological significance of bergapten in plant defense and human health research.

### 3.2. Physicochemical Properties of Bergapten: Structural Characteristics and Stability

Bergapten is a naturally occurring furanocoumarin compound belonging to the class of linear furanocoumarins characterized by a fused tricyclic structure consisting of a furan ring fused to a coumarin core (α-benzopyrone) with a methoxy substituent at the 5-position of the benzene moiety [16,40]. Bergapten’s IUPAC name is 4-methoxyfuro [3,2-g]chromen-7-one, and its molecular formula is C_12_H_8_O_4_ with a molecular weight of approximately 216.2 g/mol. The basic construction of bergapten comprises three fused rings—a benzene, a pyrone (coumarin), and a furan—resulting in a planar heterocyclic framework typical of linear furanocoumarins [40,41].

Crystallographic analysis confirms that bergapten molecules adopt an essentially planar orientation, with the oxygen-containing furan and pyrone rings aligned with minimal deviation from planarity. In the solid state, intermolecular C–H···O hydrogen bonding and π–π stacking interactions further support a tightly packed three-dimensional lattice. Bergapten is a 5-methoxy-substituted psoralen, a structural modification known in related furanocoumarins to influence photophysical and physicochemical properties [40]. Linear furocoumarins like bergapten exhibit characteristic UV absorption, which underpins many analytical detection methods (e.g., HPLC with UV detection) commonly used for quantification in plant matrices and biological samples [35]. High-performance separation methods, including HPLC and capillary electrophoresis, have been developed to separate bergapten and related furocoumarins [42].

Although detailed quantitative physicochemical data (e.g., absolute solubility values) are not routinely reported in the indexed pharmacological literature, bergapten, like other methoxylated furocoumarins, has decreased solubility in water, and modest solubility in some organic solvents [8,41,43]. Published structural studies suggest that bergapten is a stable crystalline furocoumarin under ambient laboratory conditions; however, its photostability is conditional [41]. The furan ring fused to the coumarin core readily facilitates the absorption of UVA, leading to photoactivation and potential formation of reactive intermediates capable of forming covalent adducts with pyrimidine bases in nucleic acids. Such photoadduct formation is well documented for linear furocoumarins and underlies both therapeutic photoactivation and phototoxic risk [44].

There are limited direct reports in the indexed literature quantifying bergapten’s thermal decomposition or chemical breakdown pathways under standardized stability assessment conditions. Nonetheless, its extensive use in photochemotherapy for dermatological conditions (e.g., psoriasis, vitiligo) indirectly suggests that bergapten possesses sufficient physicochemical stability for pharmaceutical formulation and systemic administration prior to photoactivation by UVA irradiation [41]. Comparative analyses of furanocoumarin derivatives indicate that structural substitution strongly influences physicochemical and photochemical properties. Hydroxylation of the coumarin nucleus is associated with increased aqueous solubility, whereas methoxylated derivatives such as bergapten are more lipophilic and exhibit reduced water solubility. Substituent-dependent differences in electronic structure, including the electron-donating character of methoxy groups, have been shown to affect excited-state behavior and photochemical reactivity in psoralen-type compounds. In parallel, variations in polarity and substitution pattern are reflected in distinct analytical and interaction profiles, including differences observed during chromatographic separation [16,43,45].

Overall, the structural, physicochemical, and photochemical characteristics of bergapten position it as a prototypical linear furanocoumarin whose behavior is governed by its rigid, planar, and highly conjugated tricyclic framework. The presence of a 5-methoxy substituent modulates both lipophilicity and electronic properties, contributing to reduced aqueous solubility, distinct chromatographic behavior, and altered excited-state dynamics relative to hydroxylated analogues. Its strong near-UV absorption, rooted in the extended π-system, underlies both analytical detectability and biologically significant photoactivity, enabling controlled therapeutic applications while also accounting for phototoxic risk. Despite limited quantitative stability and solubility data, crystallographic evidence and long-standing clinical use indicate that bergapten is chemically stable under ambient and formulation conditions, becoming reactive primarily upon UVA activation. Collectively, these integrated features illustrate how subtle structural variations within the furanocoumarin class translate into meaningful differences in physicochemical behavior, analytical handling, and photochemical reactivity, reinforcing bergapten’s relevance as both a model compound and a clinically exploited photosensitizer.

### 3.3. Pharmacokinetics of Bergapten: Evaluating the Phytochemical’s Absorption, Distribution, Metabolism, Elimination, and Toxicity

Bergapten’s intrinsic photochemical properties have historically been exploited in photochemotherapy and its diverse bioactivities—from neuroprotection to anticancer effects—have stimulated interest in its pharmacokinetic profile. However, despite numerous preclinical investigations, comprehensive human pharmacokinetic characterization remains limited [8].

#### 3.3.1. Absorption

Oral absorption of bergapten has been evaluated primarily in rodent models. After oral administration in rats, bergapten exhibits rapid gastrointestinal uptake with plasma detection within minutes and peak concentrations (T_max_) typically occurring within approximately 3–4 h, supporting its capacity for efficient gastrointestinal absorption in vivo [46]. Absolute oral bioavailability in rats has been reported to range widely but can exceed 70%, suggesting high systemic availability despite first-pass effects in the gastrointestinal tract and liver [46,47]. These findings contrast with earlier clinical observations, where serum levels of bergapten were relatively lower than those of its isomer 8-MOP, requiring higher oral doses to achieve analogous systemic exposure [48]. Detailed mechanistic studies on specific transporters mediating bergapten intestinal uptake or efflux are scarce, and its stability under variable gastrointestinal pH conditions has not been extensively quantified in the literature.

#### 3.3.2. Distribution

In rats, the volume of distribution following oral dosing is approximately 0.02 L/kg, indicating limited tissue distribution [46]. A prolonged distribution half-life (T_1/2α_) has been noted, reflective of slower equilibration between central and peripheral compartments [46]. Additionally, pharmacokinetics studies indicate that bergapten can penetrate the central nervous system and cross the blood–brain barrier [49]. The blood–brain barrier permeability likely contributes to its neuroactive effects described in preclinical models, but this distribution pattern has not been robustly quantified in humans.

#### 3.3.3. Metabolism

Hepatic biotransformation of bergapten involves both phase I and phase II enzymatic processes, as inferred from in silico predictions and limited empirical data. Computational metabolism analysis suggests the generation of multiple oxidative and conjugative metabolites through cytochrome P450-mediated oxidation and subsequent glucuronidation or sulfation reactions. These metabolites often retain or exhibit modified toxicophoric features contributing to bergapten’s toxicological profile [50]. Explicit identification of individual CYP isoforms responsible for bergapten metabolism in vivo remains an open area of investigation, though its structural similarity to related psoralens suggests that CYP-mediated biotransformation is involved [8,51,52,53]. Comprehensive characterization of major metabolites beyond in silico predictions and their pharmacological or toxicological activity is notably lacking in the literature.

#### 3.3.4. Elimination

Rodent models indicate that bergapten is eliminated through both renal and biliary pathways, with fecal excretion being the predominant route [46]. Early work in non-human primates demonstrated that a substantial fraction of administered bergapten is excreted following intramuscular or percutaneous administration, although the studies did not delineate phase II conjugate excretion profiles [54]. In rats, plasma elimination follows multi-phase kinetics, reflecting distribution and metabolic clearance contributions [46]. Specific clearance values normalized to body weight have been documented in animal models but comparable human pharmacokinetic clearance data were not identified in PubMed-indexed clinical studies.

#### 3.3.5. Toxicity and Safety Profile

Bergapten is a known photosensitizer; when administered systemically or topically and subsequently exposed to ultraviolet light, it can induce phototoxic reactions characterized by cutaneous erythema and phytophotodermatitis [48,55,56]. The phototoxicity profile has historical relevance in PUVA therapy, where 5-MOP showed reduced phototoxic side effects compared with 8-MOP in certain clinical evaluations, although its systemic absorption was lower [48,57]. Bergapten has also been associated with mutagenic potential, eliciting chromosomal aberrations consistent with DNA intercalation and crosslinking mechanisms typical of linear furocoumarins [58,59]. Computational toxicophoric analyses highlight toxic substructures shared among bergapten metabolites, underscoring the risk of metabolic activation contributing to adverse outcomes [50]. Few formal genotoxicity or hepatoxicity studies have been conducted, but preclinical evidence suggests potential dose-dependent toxicity, and drug–drug interactions mediated through hepatic enzyme modulation remain theoretically plausible given the involvement of shared metabolic pathways with other xenobiotics.

### 3.4. Implications for Therapeutic Use in Cancer Treatment

The extant pharmacokinetic literature on bergapten is predominantly based on animal studies, with only sparse human pharmacokinetic data available from early clinical photochemotherapy research. Quantitative assessment of transporter involvement, comprehensive metabolite identification in human subjects, and detailed plasma protein binding studies remain limited. Furthermore, the metabolic pathways and clearance mechanisms have not been fully delineated with regard to specific hepatic enzymes or renal transport systems, limiting the translational extrapolation of preclinical findings to potential clinical oncology applications. Notwithstanding these significant gaps in pharmacokinetic characterization, the compound’s emerging pharmacodynamic profile warrants consideration of its potential clinical applicability.

The pharmacokinetic properties of bergapten—particularly its favorable oral bioavailability in animal models and ability to penetrate the central nervous system—suggest potential utility across diverse cancer types [8,60]. However, the observed phototoxicity and incomplete metabolic characterization present challenges for its development as a chemotherapeutic or adjuvant agent [8,9]. Mechanistic anticancer effects in vitro, such as induction of autophagy and apoptosis via PTEN up-regulation and downstream Akt/mTOR pathway modulation, have been reported, indicating promising pharmacodynamic activity that may be harnessed if safety and metabolic liabilities can be properly managed [10]. Future pharmacokinetic studies in humans, incorporating detailed ADME profiling and safety assessments, are essential to determine its therapeutic index and optimize dosing strategies for oncologic applications.

A concise summary of the pharmacokinetic properties of bergapten is presented in Table 1.

## 4. Anti-Inflammatory and Antioxidant Pharmacodynamics of Bergapten: Mechanisms of Action and Therapeutic Potential

Bergapten has attracted attention for its anti-inflammatory and antioxidant bioactivities in preclinical models. A growing body of peer-reviewed, PubMed-indexed studies supports specific molecular mechanisms by which bergapten modulates inflammatory signaling, oxidative stress responses, and potentially influences carcinogenesis and therapeutic outcomes.

### 4.1. Molecular Mechanisms Underlying Anti-Inflammatory Effects

In vitro and in vivo experimental systems consistently demonstrate that bergapten attenuates classical inflammatory mediators and signaling cascades. In LPS–stimulated murine macrophages (RAW264.7 cells), bergapten significantly inhibited LPS-induced production of pro-inflammatory cytokines including TNF-α, IL-1β, and IL-6, as well as downstream inflammatory mediators such as PGE_2_ and NO through suppression of iNOS and COX-2 expression. This effect was attributed mechanistically to inhibition of JAK/STAT activation rather than direct inhibition of MAPKs or NF-κB in the specific LPS model. Furthermore, bergapten increased anti-inflammatory cytokines like IL-10 in a dose-dependent manner in these cells, indicating a bidirectional modulation of inflammatory cytokine profiles. Importantly, in vivo administration of bergapten reduced LPS-induced mortality in mice, supporting physiological relevance of these anti-inflammatory effects [61].

Other animal studies corroborate these anti-inflammatory actions. In rodent models of chemically induced inflammation and hyperalgesia, bergapten reduced expression of pro-inflammatory enzymes (iNOS and COX-2), PARP, and circulating pro-inflammatory cytokines, consistent with inhibition of key inflammatory effectors involved in nociceptive and inflammatory signaling [62].

Emerging in vivo data in a hamster model of oral squamous cell carcinoma indicate that bergapten reduces inflammatory signaling associated with tumorigenesis, including attenuation of NF-κB and PI3K/Akt/mTOR pathway activation, implicating anti-inflammatory mechanisms as part of its broader anticancer potential in preclinical carcinogenesis models [13].

Collectively, these data identify bergapten as a multi-target immunomodulatory compound that suppresses key pro-inflammatory mediators and signaling pathways while enhancing anti-inflammatory cytokine responses. Its consistent effects across in vitro and in vivo models—including reduced cytokine production, downregulation of iNOS and COX-2, attenuation of NF-κB and PI3K/Akt/mTOR signaling—support its potential relevance in inflammatory and inflammation-associated diseases.

### 4.2. Antioxidant Pharmacodynamics and Redox Regulation

Bergapten also exhibits notable antioxidant effects characterized by modulation of ROS generation, lipid peroxidation, and activation of endogenous antioxidant pathways. In LPS-stimulated macrophages, bergapten prevented ROS accumulation, an effect intimately linked with its anti-inflammatory activity given the cross-talk between ROS and inflammatory signaling cascades [61].

In rodent models of anthracycline-induced cardiotoxicity, bergapten mitigated doxorubicin-induced oxidative damage by downregulating NOX4, a principal source of intracellular ROS, and by sustaining Nrf2 expression and its downstream target HO-1. This modulation of the NOX4/Nrf2/HO-1 axis attenuated lipid peroxidation, restored redox homeostasis, and suppressed apoptosis in cardiomyocytes, illustrating a mechanistic role for Nrf2-mediated antioxidant defense in bergapten’s pharmacodynamics [63].

Additional in vivo studies of allergic airway inflammation report that bergapten increases tissue activities of canonical antioxidant enzymes such as CAT and SOD while reducing MDA, a marker of lipid peroxidation, further substantiating its capacity to enhance antioxidant defense systems in a pathophysiological context of oxidative stress [64].

The engagement of broader Nrf2/ARE pathways—including upregulation of phase II detoxifying enzymes—is consistent with established mechanisms of antioxidant phytochemicals and the documented effects of bergapten on upstream Nrf2 signaling and downstream antioxidant enzyme activities. The centrality of Nrf2 as a redox regulator, coordinating expression of SOD, CAT, and other antioxidant enzymes in response to oxidative stimuli, provides mechanistic context for interpreting bergapten’s effects within redox biology frameworks [65,66,67].

Overall, these findings support a coherent antioxidant profile for bergapten characterized by suppression of ROS-generating systems and reinforcement of endogenous cytoprotective pathways. By downregulating NOX4-dependent ROS production and sustaining Nrf2-driven transcriptional programs, bergapten limits lipid peroxidation, preserves redox homeostasis, and reduces apoptosis in oxidative stress models. Convergent increases in antioxidant enzyme activities (e.g., SOD and CAT) alongside reductions in MDA further substantiate its capacity to restore redox balance in vivo. Also, activation of the Nrf2/ARE axis provides a plausible mechanistic framework for its antioxidant effects. Collectively, these data position bergapten as a modulator of redox–inflammatory cross-talk with potential relevance in oxidative stress-associated pathologies.

### 4.3. Implications for Cancer Prevention and Therapeutic Potential

The intersecting roles of inflammation and oxidative stress in carcinogenesis make bergapten’s dual pharmacodynamic profile particularly relevant for cancer prevention and adjuvant therapy. Chronic inflammation fosters a pro-tumorigenic microenvironment through sustained cytokine production, NF-κB activation, and ROS generation, which together promote DNA damage, cell proliferation, and survival signaling [68]. Bergapten’s inhibition of inflammatory mediators (e.g., TNF-α, COX-2, iNOS) and suppression of ROS can disrupt these oncogenic processes at multiple nodes. The observed attenuation of NF-κB and PI3K/Akt/mTOR signaling in preclinical carcinogenesis models suggests that modulation of inflammation-linked survival pathways could complement conventional anticancer strategies [13].

Moreover, enhancement of antioxidant defenses via Nrf2 and endogenous antioxidant enzymes potentially ameliorates oxidative DNA damage that contributes to mutagenesis and cancer initiation. While Nrf2 activity may confer context-dependent effects in established tumors, its role in early redox homeostasis supports a chemopreventive paradigm wherein maintenance of cellular redox balance prevents malignant transformation [67,69,70].

**Figure 2:**
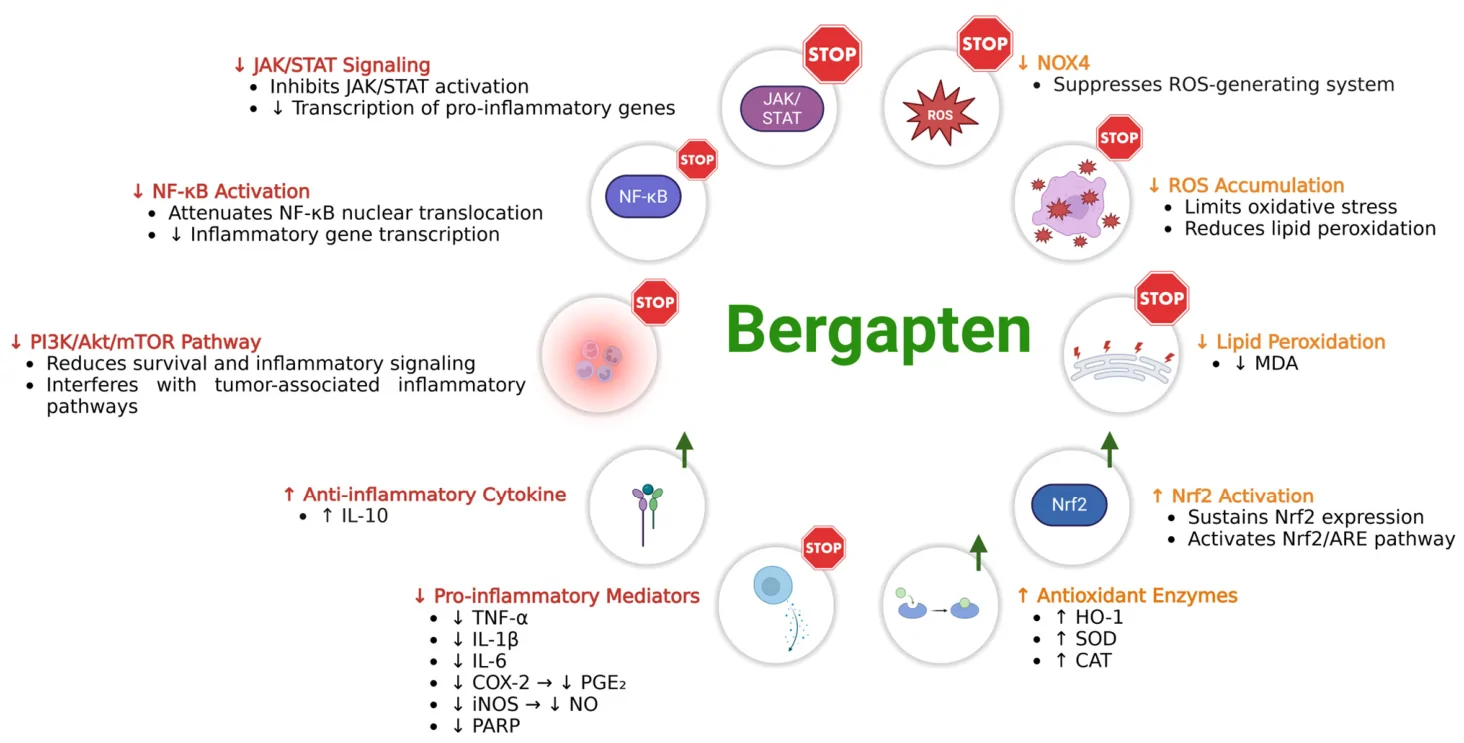
Anti-inflammatory and antioxidant pharmacodynamics of bergapten. Bergapten modulates key inflammatory signaling pathways including JAK/STAT, NF-κB, and PI3K/Akt/mTOR, leading to suppression of pro-inflammatory mediators such as iNOS and COX-2. Concurrently, it regulates redox homeostasis through activation of Nrf2-driven antioxidant responses and downregulation of NOX4-dependent ROS generation. These coordinated mechanisms contribute to immunomodulation, reduced oxidative stress, mitochondrial protection, and cytoprotective effects in preclinical models. Various symbols (↑, ↓) indicate an increase and a decrease in the obtained variables, respectively. Created in BioRender [71].

Collectively, these mechanistic insights from experimentally supported preclinical studies position bergapten as a bioactive compound with defined anti-inflammatory and antioxidant pharmacodynamics. Further investigation, including well-controlled clinical studies, will be necessary to translate these findings into potential cancer prevention or adjuvant therapeutic applications. The integrated anti-inflammatory and antioxidant pharmacodynamics of bergapten are summarized in Figure 2.

## 5. Mechanistic and Biological Axes Underlying the Anticancer Activity of Bergapten

Table 2 presents an overview of the preclinical antitumor activity of bergapten across various cancer cell lines and animal models, including concentration/exposure parameters, observed biological effects, and key molecular mechanisms. Collectively, the available preclinical evidence indicates that the antitumor activity is mediated through multiple, interconnected molecular mechanisms that converge on the regulation of cell survival, proliferation, metabolism, and therapeutic resistance. Across in vitro and in vivo models, its effects consistently involve modulation of mitochondrial apoptosis, interference with cell cycle progression, suppression of pro-survival signaling pathways, metabolic reprogramming, and, in specific contexts, enhancement of chemosensitivity or photoactivated cytotoxicity. Given this mechanistic diversity, the following sections synthesize the findings according to the principal molecular pathways involved rather than by tumor type, thereby highlighting shared and cancer-specific modes of action.

In summary, the antitumor activity emerges as a multifaceted and mechanistically coherent phenomenon characterized by the coordinated disruption of key regulatory networks governing cancer cell survival and adaptation. Across diverse tumor types, the compound consistently activates mitochondrial-dependent apoptosis, enforces cell cycle checkpoint control, suppresses PI3K/Akt/mTOR-driven survival signaling, and interferes with metabolic plasticity, particularly lipid and glycolytic pathways that sustain tumor growth. In specific biological contexts, it further promotes autophagy, reverses multidrug resistance through modulation of ABC transporters, destabilizes hormone receptor signaling, and exerts photoactivation-dependent cytotoxicity associated with DNA damage and checkpoint activation. Notably, these mechanisms are not isolated events but appear to converge on common nodal regulators such as p53, PTEN, Akt, and Bcl-2 family proteins, suggesting a systems-level reprogramming of oncogenic signaling. Collectively, the evidence supports a pleiotropic mode of action capable of targeting multiple hallmarks of cancer simultaneously, thereby providing a strong mechanistic rationale for further translational investigation and potential combinatorial therapeutic strategies.

**Figure 3:**
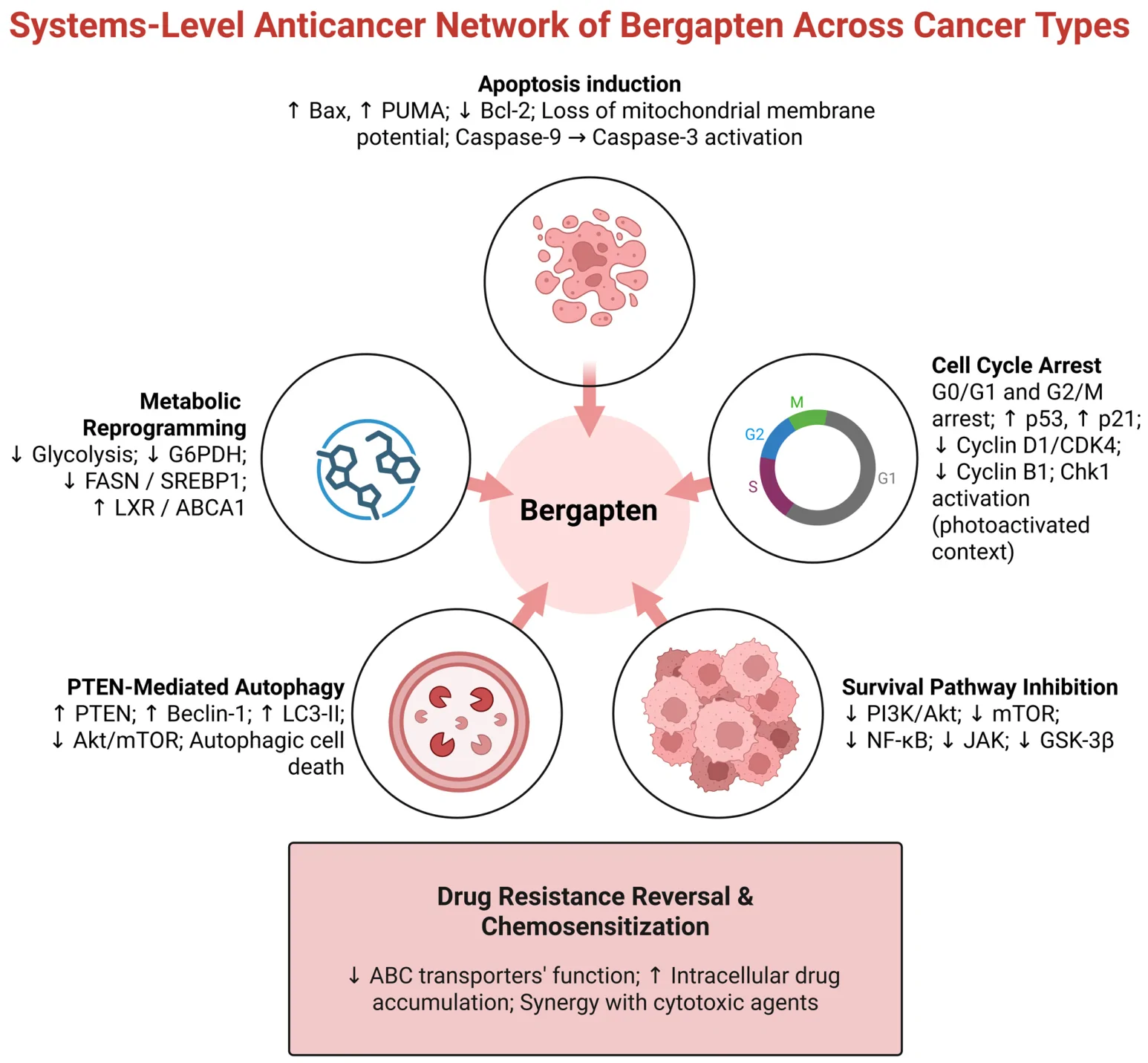
Systems-level anticancer network of bergapten across diverse cancer types. Bergapten exerts pleiotropic antitumor effects through coordinated modulation of interconnected oncogenic pathways. Central convergence on key regulatory nodes—including p53, PTEN, Akt, and Bcl-2 family proteins—leads to activation of mitochondrial-dependent apoptosis characterized by Bax upregulation, Bcl-2 suppression, and caspase activation. Bergapten enforces cell cycle arrest at G0/G1 and G2/M checkpoints via p53–p21 activation and cyclin suppression. In parallel, inhibition of PI3K/Akt/mTOR and NF-κB signaling attenuates survival and proliferative pathways. PTEN upregulation further promotes autophagic cell death through Beclin-1 and LC3-II activation. Additional mechanisms include interference with metabolic reprogramming—particularly glycolytic and lipid biosynthetic pathways—and reversal of multidrug resistance through inhibition of ABC transporters’ function, enhancing intracellular chemotherapeutic accumulation. Collectively, these integrated mechanisms highlight bergapten as a systems-level modulator of multiple hallmarks of cancer. Various symbols (↑, ↓) indicate an increase and a decrease in the obtained variables, respectively. Created in BioRender [85].

The integrated systems-level network of bergapten’s anticancer mechanisms, illustrating the convergence of apoptotic, cell cycle, survival, autophagic, metabolic, and multidrug resistance pathways on central regulatory nodes, is summarized in Figure 3.

### 5.1. Modulation of the Intrinsic Apoptotic Pathway and Mitochondrial Dysfunction

A consistent antitumor mechanism observed across multiple models is the activation of the intrinsic (mitochondrial) apoptotic pathway, characterized by modulation of Bcl-2 family proteins and caspase activation.

In oral squamous cell carcinoma, evaluated in a DMBA-induced hamster buccal pouch carcinogenesis model, oral administration (25 and 50 mg/kg for 14 weeks) significantly reduced tumor incidence and burden while increasing apoptosis. Mechanistically, treatment upregulated Bax, caspase-9, and caspase-3, while downregulating Bcl-2, indicating activation of the mitochondrial apoptotic cascade [13].

Similarly, in lung cancer models including NCI-H1975, NCI-H1299, NCI-H460, and A549 cells, bergapten reduced viability and enhanced apoptosis through increased caspase-3 activation [12,72]. Comparable findings were reported in colorectal cancer DLD-1 and LoVo cells, where 10–50 µM treatment (24–48 h) increased p53, phospho-p53, PUMA, Bax, and cleaved caspase-9 and caspase-3, confirming mitochondrial pathway engagement [74].

In thyroid cancer BCPAP cells (IC_50_ = 15 µM), apoptosis was associated with increased Bax and caspase-3/8/9 activation and reduced Bcl-2 expression [73]. In osteosarcoma Saos-2 cells, apoptosis was accompanied by loss of mitochondrial membrane potential, increased Bax, decreased Bcl-2, and activation of caspase-9 and caspase-3 [11].

In breast cancer, MCF7 and SKBR-3 cells exposed to 4–50 µM exhibited DNA fragmentation and apoptosis associated with increased caspase-8 and caspase-9 activation and p53 upregulation, further supporting both intrinsic and extrinsic pathway involvement [9].

### 5.2. Cell Cycle Arrest and Checkpoint Regulation

Cell cycle arrest represents another major mechanism underlying the antiproliferative activity observed in diverse tumor types.

In lung cancer A549 and NCI-H460 cells, treatment (10–50 µM; 24–48 h) induced G0/G1 arrest, accompanied by decreased cyclin D1 and CDK4 and increased p53, p21, and p27 expression [12]. In colorectal cancer cells, G1 arrest correlated with reduced cyclin E and CDK2 and enhanced p53–p21 axis activation [74]. Thyroid cancer cells also demonstrated reduced cyclin D1 expression [73].

In J5 cells, bergapten induced G2/M arrest, associated with cyclin B1 suppression and DNA damage [76]. In melanoma models, cell cycle perturbation was particularly evident under UVA irradiation. In B16F10 cells, exposure in combination with UVA led to G2/M arrest with increased Chk1 phosphorylation and reduced cdc2 phosphorylation [82]. In osteosarcoma Saos-2 cells, G2 arrest was likewise reported [11].

### 5.3. Inhibition of PI3K/Akt/mTOR and Related Survival Pathways

Suppression of PI3K/Akt signaling is a central mechanism linking multiple antitumor effects across cancer types.

In the DMBA-induced oral carcinogenesis model, inhibition of PI3K/Akt/mTOR and NF-κB signaling accompanied enhanced apoptosis and reduced tumor burden [13]. In lung cancer cells, downregulation of PI3K/Akt was associated with reduced migration, increased senescence, and suppression of JAK2, NF-κB, GSK-3β, TLR4, and PTK2 signaling [72]. In colorectal cancer, decreased Akt phosphorylation was accompanied by increased PTEN expression [74]. Thyroid cancer cells exhibited suppression of the PI3K/Akt/GSK-3β axis [73].

In breast cancer models, autophagy induction in MCF7 and ZR75-1 cells correlated with reduced Akt and mTOR phosphorylation and increased PTEN expression and promoter activity [10]. In osteosarcoma Saos-2 cells, decreased Akt phosphorylation was also observed [11]. In hepatocellular carcinoma in vivo, restoration of altered molecular parameters was accompanied by PI3K/Akt inhibition [75].

### 5.4. Induction of Cellular Senescence and Modulation of Inflammatory Signaling

In lung cancer models, treatment promoted cellular senescence, as evidenced by modulation of SASP factors including IL-6, IL-8, and MMP12, and of aging-related genes p16 and p21 [72]. These effects were mechanistically linked to inhibition of JAK2 and NF-κB signaling, pathways central to inflammatory and pro-survival responses.

### 5.5. Metabolic Reprogramming and Lipid Metabolism Modulation

Some studies highlighted interference with cancer metabolic pathways, particularly lipid and glucose metabolism.

In hepatocellular carcinoma models, both in vitro (HepG2 cells; EC_50_ = 269.4 and 1075 nM) and in vivo (NDEA-induced rat model; 12.5–50 mg/kg), treatment reduced lipid droplet accumulation and restored altered lipid metabolic gene expression. Mechanistically, increased LXR (α,β) expression and upregulation of ABCA1 and IDOL were accompanied by downregulation of SREBP1, LDLR, FASN, and SCD1, indicating reprogramming of lipogenic pathways [75].

In breast cancer MCF7 and ZR75-1 cells, exposure (20 and 50 µM) induced metabolic disruption characterized by reduced glycolysis, decreased G6PDH activity, altered OXPHOS complex expression, increased glucose accumulation, and enhanced lipase activity, collectively contributing to decreased cell survival [78].

### 5.6. Induction of Autophagy

Autophagic cell death was specifically demonstrated in breast cancer cells. In MCF7 and ZR75-1 models, treatment (20 and 50 µM) increased expression of Beclin-1, PI3KIII, UVRAG, and AMBRA, enhanced LC3-I to LC3-II conversion, and decreased Akt/mTOR signaling. Upregulation of PTEN further supported autophagy induction as a key antiproliferative mechanism [10].

### 5.7. Modulation of Hormone Signaling and Endocrine Resistance

In MCF7, MCF7-TR1, and ZR75 cell lines, treatment inhibited cell growth and potentiated the antiproliferative activity of OH-tamoxifen. Mechanistically, this involved modulation of the TGF-β/SMAD4 pathway, promotion of ERα degradation via the ubiquitin–proteasome system, and reduction of estrogen response gene expression [80].

### 5.8. Reversal of Multidrug Resistance via ABC Transporter Inhibition

In chemoresistant cancer models overexpressing MDR1, BCRP, or MRP2 transporters (ovarian, gastric, and breast cancer cell lines), exposure (0–100 µM) enhanced intracellular accumulation and cytotoxicity of daunorubicin, mitoxantrone, and cisplatin. These effects were mediated by modulation of ABC transporter gene expression and inhibition of transporter function, indicating a chemosensitizing role [84].

### 5.9. Photoactivation-Dependent Cytotoxicity

In melanoma models (C32, COLO829, A375, and B16F10), cytotoxicity was markedly enhanced under UV irradiation. EC_50_ and IC_50_ values were substantially reduced following UV exposure [81,82,83]. Although detailed molecular pathways were not fully elucidated in all studies, G2/M arrest and checkpoint activation were demonstrated in B16F10 cells, supporting DNA damage-mediated cytotoxic mechanisms under photoactivation conditions.

### 5.10. Studies Without Mechanistic Elucidation

In certain breast cancer and melanoma models, reductions in viability, proliferation, or migration were reported; however, the molecular mechanisms underlying these effects were not experimentally characterized [77,79,81,83].

## 6. Mechanistic Insights into the Antitumor Activity of Bergapten Derivatives and Bergapten-Containing Formulations

Table 3 summarizes the preclinical antitumor activity, experimental models, and underlying molecular mechanisms of bergapten, its derivatives, and formulations across diverse cancer types. The preclinical evidence presented in these studies demonstrates that bergapten, its derivatives, and bergapten-containing formulations exhibit significant antitumor activity across multiple cancer models, including pancreatic, breast, and neuroblastoma. In pancreatic cancer, FSG-Y5—a bergapten derivative—showed markedly enhanced potency relative to bergapten, significantly inhibiting CFPAC-1 and PANC-1 cell proliferation, migration, and organoid survival, while inducing apoptosis through upregulation of Bax and cleaved caspase-3 [14]. In vivo, FSG-Y5 reduced tumor growth and metastasis in zebrafish embryo models, highlighting its translational potential, although molecular mechanisms in vivo were not detailed.

Similarly, bergapten-containing extracts and formulations demonstrated promising antitumor activity. The WSZG extract, administered intragastrically in a breast cancer bone metastasis xenograft model, prevented body weight loss, reduced the incidence and severity of metastases, and decreased tumor cell infiltration and osteolytic lesions [86]. In neuroblastoma SH-SY5Y cells, liposomal BEO increased cytotoxicity compared with non-liposomal BEO, indicating that formulation strategies can enhance cellular uptake and antitumor efficacy [87].

Collectively, these studies highlight several important mechanistic and translational themes. First, induction of apoptosis via mitochondrial pathways appears to be a key mediator of cytotoxicity, as evidenced by Bax upregulation and caspase-3 activation in pancreatic cancer cells [14]. Second, chemical modification of bergapten can substantially improve potency, while formulation strategies, such as liposomal encapsulation, enhance bioavailability and cellular delivery [86,87]. Third, although in vitro mechanisms are often well characterized, most in vivo studies did not report molecular endpoints, representing an important knowledge gap for translational development.

These findings collectively suggest that bergapten derivatives and bergapten-containing formulations possess pleiotropic antitumor properties, capable of targeting multiple hallmarks of cancer, including proliferation, migration, survival, and metastasis. Future research should aim to elucidate in vivo molecular mechanisms, optimize dosing regimens, and explore combinatorial strategies with existing chemotherapeutics to maximize clinical relevance. Together, these studies provide a strong rationale for further preclinical and translational investigation of bergapten-based therapies [14,86,87].

## 7. Limitations of the Available Evidence

While the preclinical studies summarized in Table 2 and Table 3 provide compelling evidence of the antitumor potential of bergapten, its derivatives, and formulations, several important limitations should be considered. First, most studies rely heavily on in vitro models, with a substantial proportion of experiments conducted in standard cancer cell lines. Although these models allow for controlled mechanistic investigations, they may not fully recapitulate the complex tumor microenvironment, intercellular interactions, or pharmacokinetics observed in vivo. Consequently, extrapolation of efficacy from cell culture to whole organisms remains uncertain.

Second, although many in vivo studies demonstrate reductions in tumor growth and metastasis, molecular mechanisms were frequently not assessed in these models. This gap limits our understanding of how bergapten or its derivatives interact with systemic physiology, tissue-specific metabolism, and host immune responses. In addition, dosing regimens and exposure durations vary widely across studies, making it difficult to establish standardized effective concentrations or to compare results quantitatively between models.

Third, although derivatives (e.g., FSG-Y5) and formulations (e.g., liposomal BEO) show enhanced efficacy, there is a lack of long-term toxicity and pharmacokinetic data, which are critical for translational development. Furthermore, many studies focus on single-agent interventions, and few assess potential synergistic or antagonistic interactions with established chemotherapeutics. Finally, the diversity of cancer types, cell lines, and assay endpoints contributes to heterogeneity, limiting the generalizability of findings and complicating systematic meta-analyses.

In summary, while preclinical evidence supports the antitumor promise of bergapten-based interventions, the field would benefit from more comprehensive in vivo mechanistic studies, standardized dosing protocols, toxicity and pharmacokinetic evaluations, and exploration of combination therapies to strengthen translational relevance.

### Comparative Dose–Response Analysis Across Preclinical Models

A notable limitation across the preclinical literature on bergapten is the absence of an integrated quantitative comparison of effective concentrations and dose–response relationships among experimental systems. Although numerous studies report concentration ranges in micromolar units for in vitro experiments and dosing regimens in mg/kg for in vivo models, these parameters are generally presented descriptively rather than comparatively. A cross-model dose–response synthesis provides a more coherent quantitative framework for interpreting relative potency, contextual sensitivity, and structure–activity relationships.

Across diverse tumor cell lines, the most frequently reported concentrations for non-photoactivated bergapten were at or below 50 µM. An exception to this micromolar pattern is observed in certain hepatocellular carcinoma models, where nanomolar EC_50_ values have been reported in HepG2 cells [75]. This apparent increased sensitivity may reflect tumor-specific metabolic vulnerability or assay-dependent differences and warrants further validation under standardized comparative conditions.

In melanoma models subjected to UV irradiation, the concentration required for cytotoxicity decreases substantially compared with non-irradiated conditions, with markedly lower EC_50_ or IC_50_ values following photoactivation [81,82,83]. These findings confirm bergapten’s classical furanocoumarin photosensitizing properties and demonstrate that its dose–response behavior is strongly modulated by light exposure.

In vivo dosing paradigms across rodent and hamster carcinogenesis models most commonly range between 12.5 and 50 mg/kg [13,75]. Oral administration at 25 and 50 mg/kg in the DMBA-induced oral squamous cell carcinoma model significantly reduced tumor incidence and modulated PI3K/Akt/mTOR signaling while increasing apoptotic markers [13]. Similarly, hepatocellular carcinoma models treated with 12.5–50 mg/kg demonstrated metabolic reprogramming and antitumor effects [75]. However, few studies provide plasma exposure data or pharmacokinetic–pharmacodynamic correlations, limiting quantitative bridging between in vitro IC_50_ values and achievable systemic concentrations.

Comparative analysis of structurally modified derivatives further contextualizes bergapten’s potency. The derivative FSG-Y5, evaluated in pancreatic cancer models including CFPAC-1 and PANC-1 cells, demonstrated enhanced antiproliferative effects relative to the parent compound [14]. Experimental evidence indicates stronger inhibition of proliferation and migration, greater induction of Bax and cleaved caspase-3 expression, and significant suppression of organoid viability. In zebrafish embryo xenograft systems, FSG-Y5 also significantly reduced tumor growth and metastatic dissemination [14]. Although direct side-by-side IC_50_ comparisons under identical experimental conditions remain limited, the available data consistently suggest superior potency of FSG-Y5 relative to bergapten, supporting ongoing structure–activity optimization of the psoralen scaffold.

Taken together, the dose–response profile of bergapten across preclinical models can be summarized as follows: a typical in vitro concentration window at or below 50 µM and in vivo activity predominantly observed at doses at or below 50 mg/kg. Photoactivation substantially lowers effective cytotoxic concentrations in melanoma models [81,82,83], while derivative development, exemplified by FSG-Y5, enhances intrinsic potency [14]. This integrated quantitative perspective strengthens the translational interpretation of the preclinical data and highlights the need for standardized pharmacokinetic–pharmacodynamic modeling to determine whether therapeutically achievable systemic concentrations can approximate the effective in vitro concentration range observed across tumor types.

## 8. Translational Challenges and Future Clinical Development

Despite extensive mechanistic and preclinical evidence supporting the biological activity of bergapten, there remains a substantial gap between these findings and potential clinical application. Bergapten possesses similar therapeutic potential to 8-MOP, a compound that has been used clinically for decades in PUVA therapy to treat dermatological conditions such as psoriasis and vitiligo [8,48]. While 8-MOP has a well-characterized clinical and pharmacokinetic profile in dermatologic contexts, bergapten has not been systematically evaluated beyond historical and limited dermatological uses. This absence of robust clinical data, particularly in oncology, poses a challenge to translational development. The structural and mechanistic similarities between bergapten and 8-MOP provide a framework for regulatory familiarity but also introduce concerns related to class-associated risks, notably phototoxicity and potential long-term mutagenicity.

Another critical translational issue concerns the relationship between in vitro effective concentrations and clinically achievable systemic exposure. Antiproliferative, pro-apoptotic, and anti-migratory effects of bergapten have been reported in a range of in vitro tumor models at micromolar concentrations [11]. However, human pharmacokinetic studies of psoralens, including 8-MOP, demonstrate that systemic exposure is influenced by dose, and that peak plasma concentrations decline rapidly following administration [88]. Without analogous pharmacokinetic data for bergapten in humans, the translational relevance of in vitro potency remains uncertain. Establishing whether systemic exposures that correlate with in vitro efficacy can be safely achieved is essential before clinical trials can be justified.

Phototoxicity represents another translational barrier. Furanocoumarins form DNA adducts upon absorption of UVA radiation, a mechanism that is exploited therapeutically in controlled PUVA protocols but that also underlies phototoxic reactions and potential damage in non-target tissues [16,89]. Although bergapten has been associated with lower phototoxic responses compared to 8-MOP in some dermatological studies, the risk of phototoxicity with systemic administration persists and necessitates careful evaluation in preclinical safety studies [48,57]. These investigations should quantify the extent of light-induced DNA damage in relevant tissues and assess strategies for mitigating phototoxic risk, such as shielding or modified drug delivery systems.

Beyond phototoxic effects, evidence of mutagenicity and clastogenicity associated with furanocoumarins warrants thorough safety assessment. Early genotoxicity studies demonstrated that bergapten and related compounds can induce mutagenic and clastogenic effects in bacterial and cellular assays [90]. The potential for genotoxicity raises long-term safety concerns that must be addressed through standardized genotoxic and carcinogenicity testing protocols consistent with regulatory expectations for agents under consideration for oncologic use.

From a regulatory perspective, furanocoumarins are subject to scrutiny due to their photobiological activities and potential health risks associated with environmental and dietary exposures. Any proposal to reposition bergapten as a systemic anticancer agent will require a comprehensive safety dossier, including acute and chronic toxicity studies, genotoxicity assessments, and pharmacokinetic characterization in humans. Regulatory authorities such as the U.S. Food and Drug Administration and the European Medicines Agency are likely to require evidence that systemic exposures can be managed safely and that risks such as phototoxicity and mutagenicity are sufficiently mitigated.

To bridge the translational gap, several strategic pathways should be pursued. First, detailed human pharmacokinetic studies are needed to determine whether therapeutic plasma concentrations are achievable without intolerable toxicity. Such studies could evaluate alternative formulations or delivery methods that enhance bioavailability while minimizing systemic exposure to light-sensitive tissues. Second, a comprehensive suite of safety and genotoxicity studies should be conducted in accordance with regulatory guidelines to define the risk profile of bergapten. Third, translational models that better emulate human tumor biology, such as patient-derived xenografts and organotypic cultures, should be employed to refine exposure–response relationships and inform dose selection. Finally, if photodynamic mechanisms are to be harnessed therapeutically, controlled strategies that localize light delivery to the tumor microenvironment should be explored to maximize efficacy while minimizing off-target effects.

In summary, although bergapten exhibits promising anticancer activity in preclinical models, substantial translational challenges remain. Addressing these challenges through rigorous pharmacokinetic, safety, and mechanistic studies will be critical to progress from promising laboratory findings toward clinical evaluation. A translationally oriented research agenda that integrates comparative pharmacology, exposure assessment, and risk mitigation will elevate the clinical relevance of bergapten and clarify its potential role in therapeutic development.

## 9. Perspectives on Nanotechnology-Enabled Delivery Strategies for Bergapten in Cancer Therapy

Despite compelling mechanistic and anticancer evidence for bergapten, its physicochemical profile—notably poor aqueous solubility and limited biodistribution—poses barriers to effective systemic delivery and clinical translation [8,91,92,93]. These challenges mirror those encountered with many phytochemicals, where low solubility and rapid clearance restrict therapeutic exposure at target tissues, necessitating innovative formulation strategies to optimize pharmacokinetics, bioavailability, and tumor targeting.

A growing body of research in related natural anticancer agents highlights the potential of nanocarrier platforms—including liposomes, SLNs, polymeric micelles, and other nanostructured systems—to enhance delivery of poorly soluble bioactive compounds. Nanocarriers can improve dissolution, stabilize labile compounds, enable controlled release, and facilitate accumulation in tumor tissues via EPR effects [91,94,95]. Specifically for bergapten, formulation into lipid-based nanomedicines has begun to emerge as a promising strategy: in an inhalable lipid-nanomedicine formulation, bergapten incorporated into DPPC liposomes (‘Ber-lipo’) demonstrated good stability and target engagement in vivo, suggesting that phospholipid vesicles can serve as an effective delivery vehicle for this phytochemical [93].

Lipid carriers such as liposomes and nanostructured lipid carriers are particularly attractive for furanocoumarins like bergapten due to their ability to incorporate lipophilic drugs within the lipid bilayer or matrix, improving solubility and biodistribution. Although most studies to date have focused on anti-inflammatory applications, the fundamental principles apply directly to oncology, where enhanced delivery to tumor or inflammatory microenvironments is critical [93,94,96].

Alternative lipidic structures, such as monoolein-based cubosomes, have been examined for sustained release of bergapten, indicating that controlled release from structured lipid assemblies could mitigate rapid redistribution and improve therapeutic window—a principle that is highly relevant to anticancer delivery systems where prolonged exposure may enhance cytotoxicity [97].

While direct nanoencapsulation of bergapten in anticancer applications remains in early stages, broader literature on coumarin derivatives supports the concept that nanoformulations can potentiate anticancer activity by improving pharmacokinetic behavior and cellular uptake. Reviews of nanoformulated coumarins demonstrate that this class of compounds—including furanocoumarins related to bergapten—is amenable to diverse nanocarrier platforms that enhance stability, solubility, and tumor targeting while potentially enabling co-delivery with conventional chemotherapeutics to overcome drug resistance or reduce systemic toxicity [91,98,99,100,101].

Future formulation efforts should integrate passive and active targeting mechanisms—such as ligand-functionalized nanoparticles or pH-responsive systems—to increase bergapten’s tumor selectivity and minimize off-target effects. Such strategies could be adapted for bergapten to leverage its multi-targeted anticancer mechanisms while addressing bioavailability limitations [94,102].

**Figure 4:**
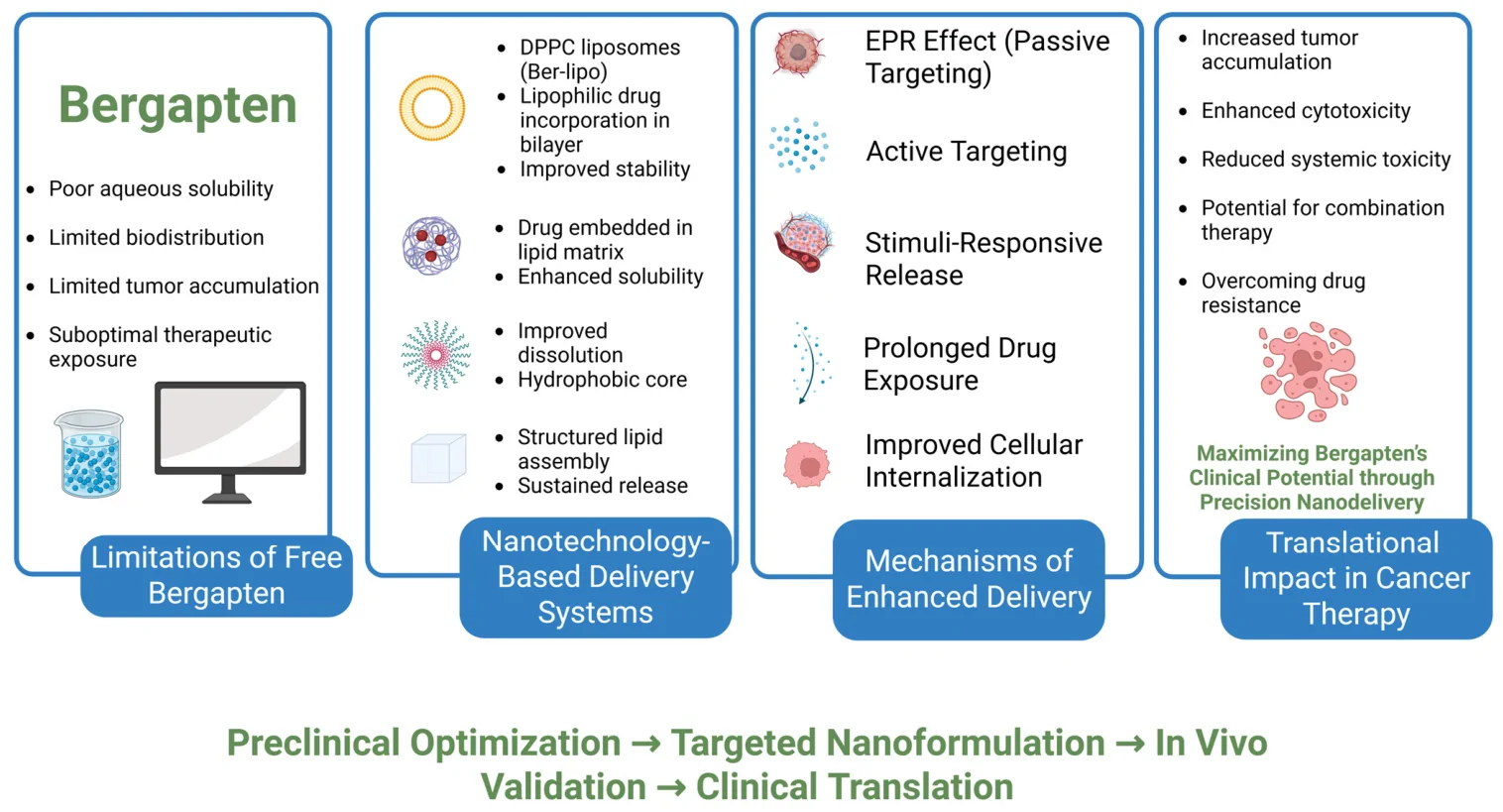
Schematic representation of nanotechnology-enabled delivery strategies to enhance bergapten bioavailability and tumor targeting in cancer therapy. Lipid-based and polymeric nanocarriers improve solubility, stability, controlled release, and tumor accumulation via passive and active targeting mechanisms, thereby enhancing therapeutic efficacy while minimizing systemic toxicity. Created in BioRender [103].

Overall, these formulation approaches underscore the translational imperative for optimizing bergapten delivery to achieve appropriate systemic levels, controlled release, and enhanced accumulation at tumor sites. Advancing validated nanocarrier designs—from lipid vesicles to structured nanoparticles—will be crucial to realize bergapten’s clinical potential in cancer therapy. The possible nanotechnology-enabled strategies to enhance bergapten delivery are summarized in Figure 4.

## 10. Conclusions

Bergapten emerges from the current body of evidence as a pharmacologically versatile furanocoumarin with significant anticancer promise supported by mechanistic depth across diverse experimental systems. Derived predominantly from Rutaceae and Apiaceae plant families and biosynthesized through the phenylpropanoid–furanocoumarin axis, bergapten possesses well-defined structural and photochemical properties that underlie both its therapeutic utility and its safety considerations. Its planar, conjugated tricyclic scaffold confers near-UV absorbance and biological reactivity, while its methoxy substitution modulates lipophilicity, metabolic behavior, and photochemical properties.

Preclinical studies consistently demonstrate that bergapten exerts pleiotropic antitumor effects across multiple cancer models. Mechanistically, its anticancer activity converges on several hallmark regulatory axes: (i) activation of mitochondrial-dependent apoptosis via modulation of Bax/Bcl-2 balance and caspase cascades; (ii) enforcement of cell cycle checkpoints at G0/G1 or G2/M phases through p53–p21 signaling and cyclin suppression; (iii) inhibition of pro-survival pathways such as PI3K/Akt/mTOR and NF-κB; (iv) induction of autophagy linked to PTEN upregulation and Akt/mTOR attenuation; (v) interference with metabolic reprogramming, particularly lipid and glycolytic pathways; and (vi) reversal of multidrug resistance through modulation of ABC transporters. In hormone-responsive tumors, additional regulation of ERα signaling and TGF-β/SMAD pathways has been described. Collectively, these coordinated actions suggest that bergapten functions not as a single-target cytotoxic agent but as a systems-level modulator of oncogenic signaling networks.

Beyond direct cytotoxicity, bergapten’s anti-inflammatory and antioxidant pharmacodynamics further reinforce its relevance in oncology. By attenuating JAK/STAT, NF-κB, and PI3K/Akt/mTOR signaling and reducing pro-inflammatory mediators (e.g., TNF-α, IL-6, COX-2, iNOS), while simultaneously sustaining Nrf2-driven antioxidant defenses and limiting NOX4-dependent ROS production, bergapten targets the redox–inflammatory interface central to tumor initiation and progression. This dual activity supports a potential chemopreventive role in inflammation-associated carcinogenesis and a complementary role in mitigating oxidative injury during anticancer therapy.

Despite these encouraging findings, translation into clinical oncology remains at an early stage. The current evidence base is dominated by in vitro investigations, with comparatively limited in vivo mechanistic validation and scarce human pharmacokinetic data. Key translational gaps include incomplete characterization of metabolic pathways, limited understanding of plasma protein binding and transporter interactions, insufficient long-term toxicity assessment, and heterogeneity in dosing paradigms across studies. Phototoxicity, while therapeutically exploitable in controlled settings, also necessitates careful risk management for systemic applications.

Advances in medicinal chemistry and formulation science provide promising avenues to overcome these limitations. Bergapten derivatives such as FSG-Y5 demonstrate enhanced potency in pancreatic cancer models, and lipid-based or nanoengineered delivery systems offer strategies to address poor aqueous solubility, optimize biodistribution, and improve tumor targeting. Nanocarrier platforms—including liposomes, structured lipid nanoparticles, and polymeric systems—may enable controlled release, co-delivery with chemotherapeutics, and reduction in off-target effects, thereby enhancing therapeutic index. However, robust in vivo pharmacodynamic validation, standardized ADME profiling, and rigorous safety evaluation are essential prerequisites for clinical advancement.

In conclusion, bergapten represents a mechanistically multifaceted phytochemical with the capacity to modulate multiple hallmarks of cancer simultaneously. Its integrated effects on apoptosis, cell cycle regulation, survival signaling, metabolic adaptation, inflammation, and oxidative stress provide a compelling biological rationale for continued translational development. Future research should prioritize comprehensive pharmacokinetic characterization in humans, systematic in vivo mechanistic studies, optimization of nanoenabled delivery systems, and exploration of rational combination regimens with established anticancer agents. Through such integrative efforts, bergapten may progress from a promising preclinical compound to a clinically relevant adjunct or therapeutic candidate in oncology.

## Figures and Tables

**Table 1:** Simplified Summary of Bergapten Pharmacokinetics.

Parameter	Summary
Absorption	Rapid oral absorption in rats; T_max_: ~3–4 h; bioavailability may exceed 70%. Lower systemic exposure observed in humans vs. 8-MOP.
Distribution	Low volume of distribution (~0.02 L/kg in rats); limited tissue accumulation; capable of crossing the blood–brain barrier.
Metabolism	Undergoes phase I (primarily CYP-mediated) and phase II (conjugation) metabolism; data mainly predicted or limited.
Elimination	Predominantly excreted via feces; renal and biliary routes; multi-phase kinetics.
Toxicity	Phototoxic (UV-induced skin reactions); potential mutagenicity; possible dose-dependent toxicity; limited in vivo safety data.
Overall limitations	Data predominantly from animal and in silico studies; human pharmacokinetic data remain scarce and incomplete.

Note. Data derived from Refs. [8,9,10,46,47,48,49,50,51,52,53,54,55,56,57,58,59,60].

**Table 2:** Preclinical Evidence of Antitumor Activity and Mechanisms of Action of Bergapten Across Cancer Models.

Cell Line (s)/Animal Model (s)	IC_50_/EC_50_/Concentration and Exposure Time	Main Antitumor Effects	Key Molecular Mechanisms	Reference
Oral squamous cell carcinoma				
In vivo: DMBA-induced hamster buccal pouch carcinogenesis model.	In vivo: 25 and 50 mg/kg (oral administration) for 14 weeks.	In vivo: altered the levels of lipid peroxidation, antioxidant, and xenobiotic metabolic enzymes, ↓ body weight loss, ┴ tumor incidence and tumor burden, ↑ apoptosis.	In vivo: ↑ Bax, caspase-9 and caspase-3; ↓ Bcl-2; ┴ PI3K/Akt/mTOR and NF-κB signaling.	[13]
Lung cancer				
In vitro: NCI-H1975, NCI-H1299 and NCI-H460 cell lines.	In vitro: 10, 20, 30, 40, 50 and 60 µM; 12, 24 and 72 h.	In vitro: promotion of cellular senescence, ↓ cell viability and migration, ↑ apoptosis.	In vitro: modulation of SASP factors (IL-6, IL-8, MMP12) and aging-related genes (p16 and p21); ↑ caspase-3; ┴ cyclin D1, JAK2, NF-κB, GSK-3β, TLR4, and PTK2; ┴ PI3K/Akt pathway.	[72]
In vitro: A549 and NCI-H460 cell lines.	In vitro: 10, 20, 30, 40 and 50 µM; 24 and 48 h.	In vitro: ↓ cell viability, ↑ apoptosis.	In vitro: induction of G0/G1 cell cycle arrest; ↓ cyclin D1 and CDK4; ↑ p53, p21 and p27; ↑ Bax and cleavage of caspase-3; ↓ Bcl-2.	[12]
Thyroid cancer				
In vitro: BCPAP cell line.	In vitro: IC_50_ = 15 µM.	In vitro: ┴ cell proliferation and viability, ↑ apoptosis.	In vitro: ↑ Bax, caspase-3/8/9; ↓ Bcl-2, cyclin D1, c-Myc, and surviving; ┴ PI3K/Akt/GSK-3β.	[73]
Colorectal cancer				
In vitro: DLD-1 and LoVo cell lines.	In vitro: 10, 30 and 50 µM; 24 and 48 h.	In vitro: ↓ cell viability, ↑ apoptosis.	In vitro: induction of cell cycle arrest at G1; ↑ p53, phospho-p53, p21, PUMA, Bax, PTEN, and the caspase-9 and caspase-3 cleavage; ↓ cyclin E, CDK2, and p-Akt.	[74]
Liver cancer				
In vitro: HepG2 cell line. In vivo: NDEA-induced liver cancer in Wistar rats.	In vitro: EC_50_ = 269.4 nM and EC_50_ = 1075 nM. In vivo: 12.5, 25 and 50 mg/kg (intraperitoneal injection) for 2 weeks.	In vitro: ↓ lipid droplets accumulation (controlled carcinogenesis by altering lipogenic pathways). In vivo: ameliorated the cancer induced alterations in body weight and liver weight, ↓ lipid accumulation in carcinoma tissue (controlled carcinogenesis by altering lipogenic pathways).	In vitro: ↑ LXR (α,β). In vivo: restoration of the changes in mRNA and protein expressions (↓ SREBP1, LDLR, FASN and SCD1, ↑ LXR (α,β), ABCA1 and IDOL); ┴ PI3K/Akt pathway.	[75]
In vitro: J5 cell line.	In vitro: 0.05, 0.5, 5, 10, 25 and 50 mM; 12, 24, 48 and 72 h.	In vitro: induction of damage in cell morphology, ↓ cell viability and proliferation, ↑ apoptosis.	In vitro: promotion of DNA damage; induction of cell cycle arrest at G2/M; ┴ cyclin B1.	[76]
Breast cancer				
In vitro: MDA-MB-231 cell line.	In vitro: 5, 10, 15, 20, 25 and 30 μg/mL; 24, 48 and 72 h.	In vitro: ↓ cell viability and proliferation, ┴ cell migration.	In vitro: molecular mechanisms were not experimentally evaluated and specified.	[77]
In vitro: MCF7 and ZR75-1 cell lines.	In vitro: 20 and 50 μM; 6 and 16 h.	In vitro: induction of metabolic reprogramming and cell death, promotion of lipid-lowering effect, ↓ cell survival, ┴ glycolysis, ↑ glucose accumulation.	In vitro: modulation of the expression of OXPHOS complexes and ATP production; ↓ G6PDH activity; ↑ lipase activity.	[78]
In vitro: MCF7 and MDA-MB-231 cell lines.	In vitro: 5, 10, 20 and 50 μM; 3, 6, 12, 18, 24 and 48 h.	In vitro: ↓ cell viability and proliferation.	In vitro: molecular mechanisms were not experimentally evaluated and specified.	[79]
In vitro: MCF7 and ZR75-1 cell lines.	In vitro: 20 and 50 μM; 6, 10, 16, 24 and 48 h.	In vitro: induction of autophagy, ↓ cell viability and survival.	In vitro: upregulation of PTEN expression and PTEN gene promoter activity; ↑ Beclin-1, PI3KIII, UVRAG, AMBRA expression; ↑ conversion of LC3-I to LC3-II; ↓ p-Akt, Akt, p-mTOR, mTOR expression levels.	[10]
In vitro: MCF7, Tamoxifen-resistant MCF7-TR1 and ZR75 cell lines.	In vitro: 5, 10, 20 and 50 μM; 2, 4, 6, 24, 48 and 96 h.	In vitro: ┴ cell growth, ↑ antiproliferative activity of OH-Tamoxifen.	In vitro: modulation of TGF-β pathway and SMAD4 protein expression (in MCF7 and MCF7-TR1 cells); promotion of ERα degradation via the ubiquitin-proteasome pathway; downregulation of ERα protein content; ↓ estrogen response gene expression.	[80]
In vitro: MCF7 and SKBR-3 cell lines.	In vitro: 4, 6, 10, 20 and 50 μM; 5–30 min, 1, 2, 6, 12, 24 and 48 h.	In vitro: induction of DNA fragmentation, ↑ apoptosis.	In vitro: transactivation of p53 gene promoter; induction of cell cycle arrest at G0/G1; ↑ p53 and p21 mRNA and protein levels; ↑ caspase-8 and caspase-9 activation; ↑ NF-Y nuclear translocation; ↑ p38 MAPK activation.	[9]
Melanoma				
In vitro: C32 and COLO829 cell lines.	In vitro: for C32 cells, EC_50_ = 560.6 μM (bergapten alone), 22.7 μM (after 30 min exposure to UVA radiation) and 24.2 μM (after 60 min of UVA radiation); for COLO829 cells, EC_50_ = 39.4 μM (bergapten alone), 7.9 μM (after 30 min exposure to UVA radiation) and 7 μM (after 60 min of UVA radiation).	In vitro: ↓ cell viability; cytotoxic response was significantly augmented in cells exposed to UVA radiation.	In vitro: molecular mechanisms were not experimentally evaluated and specified.	[81]
In vitro: B16F10 cell line. In vivo: B16F10 melanoma bearing mice under UVA irradiation.	In vitro: IC_50_ = 0.06 µM (in UVA-irradiated cells). In vivo: 0.5 or 1.0 mg/kg (intraperitoneal injection) daily for 20 consecutive days.	In vitro: ┴ cell growth and proliferation; in the absence of UVA irradiation, bergapten had no effect. In vivo: ↓ tumor growth and final tumor weight.	In vitro: induction of cell cycle arrest at G2/M; ↑ Chk1 phosphorylation; ↓ cdc2 phosphorylation. In vivo: molecular mechanisms were not experimentally evaluated.	[82]
In vitro: C32 and A375 cell lines.	In vitro: IC_50_ = 71.7 ± 2.9 µg/mL (after 20 min exposure to UV irradiation), 51.64 ± 2.1 µg/mL (after 40 min), 32.51 ± 1.8 µg/mL (after 60 min), 9.1 ± 0.6 µg/mL (after 100 min); all these values are for A375 cells.	In vitro: ┴ cell proliferation (only A375 cells); bergapten cytotoxicity was induced by UV irradiation.	In vitro: molecular mechanisms were not experimentally evaluated and specified.	[83]
Studies evaluating multiple cancers				
In vitro: RPMI 8226 and U266 cell lines (multiple myeloma), HT-29 and SW620 cell lines (colon cancer), Saos-2 and HOS cell lines (osteosarcoma).	In vitro: IC_50_ = 40.05 µM (Saos-2); IC_50_ = 332.4 µM (HT-29); IC_50_ = 354.5 µM (SW620); IC_50_ = 257.5 µM (HOS); IC_50_ = 1272 µM (RPMI 8226); IC_50_ = 1190 µM (U266).	In vitro: ↓ cell viability and proliferation (principally in colon cancer and osteosarcoma cell lines), ↓ cell migration (principally in Saos-2 and HT-29), ↑ apoptosis (in Saos-2).	In vitro: induction of cell cycle arrest at G2; loss of mitochondrial membrane potential; ↑ Bax; ↓ Bcl-2; ↑ caspase-9 and caspase-3 activation; ↓ Akt phosphorylation (all these mechanisms were observed in Saos-2 cells).	[11]
In vitro: A2780RCIS (MRP2 overexpressing ovarian cancer cell line), EPG85.257RDB (MDR1 overexpressing gastric cancer cell line), MCF7MX (BCRP overexpressing breast cancer cell line) and their parental–A2780, EPG85.257 and MCF7–lines.	In vitro: 0–100 μM; 24, 48, 72 h and 5 days.	In vitro: ↓ cell viability, ↑ cytotoxicity of daunorubicin, mitoxantrone, and cisplatin, and intracellular accumulation of chemotherapeutics (in the resistant cell lines).	In vitro: modulation of ABC transporter gene expression; ┴ MDR1, BCRP, and MRP transporter function.	[84]

Note. Various symbols (↑, ↓, and ┴) indicate an increase, a decrease, and an inhibition in the obtained variables, respectively.

**Table 3:** Preclinical Antitumor Activity and Molecular Mechanisms of Bergapten Derivatives and Bergapten-Containing Formulations Across Cancer Models.

Cell Line (s)/Animal Model (s)	IC_50_/EC_50_/Concentration and Exposure Time	Main Antitumor Effects	Key Molecular Mechanisms	Reference
Bergapten derivatives				
In vitro: human pancreatic cancer lines (SW1990, Capan-2, CFPAC-1 and PANC-1) and pancreatic cancer organoids. In vivo: tumor (CFPAC-1 and PANC-1 cells)-bearing zebrafish embryos.	In vitro: 5, 10, 20 μM; 6, 24 and 48 h (FSG-Y5). Bergapten and other FSG-Y derivatives in concentrations ranging from 0.05 to 50 µmol/L. In vivo: 5, 10, and 20 μM; 48 h (FSG-Y5).	In vitro: ┴ cell proliferation and survival, ↑ apoptosis, ┴ cell migration (all these effects were observed in CFPAC-1 and PANC-1 cells after FSG-Y5 treatment; FSG-Y5 significantly inhibited CFPAC-1 and PANC-1 cells by ~12 and ~7 times compared with bergapten, respectively), ┴ organoid survival. In vivo: ┴ tumor growth and metastasis.	In vitro: ↑ Bax and cleaved caspase-3 (in CFPAC-1 and PANC-1 cells after FSG-Y5). In vivo: molecular mechanisms were not specified.	[14]
Bergapten formulations				
In vivo: xenograft model of breast cancer bone metastases (inoculation of MDA-MB-231BO cells in BALB/c nude mice).	In vivo: WSZG extract (intragastric gavage) 1.6 g/kg, equivalent to 2.73 mg/kg bergapten daily, for 4 weeks.	In vivo: prevented the loss of body weight, ┴ the intensity and incidence of bone metastasis, ↓ tumor cell infiltration and osteolytic lesion in bone.	In vivo: molecular mechanisms were not specified.	[86]
In vitro: SH-SY5Y human neuroblastoma cells.	In vitro: BEO and BEO-loaded liposomes (0.01%, 0.02%, 0.03%, 0.04% and 0.05% *v*/*v*); 24, 48 and 72 h.	In vitro: ↑ cell mortality, ↓ cell viability; BEO and BEO-BF liposomes showed superior anticancer activity compared with their non-liposomal forms; BEO-BF was more efficacious than BEO in both non-liposomal and encapsulated forms.	In vitro: molecular mechanisms were not specified.	[87]

Note. Various symbols (↑, ↓, and ┴) indicate an increase, a decrease, and an inhibition in the obtained variables, respectively.

## Data Availability

No new data were created or analyzed in this study. Data sharing is not applicable to this article.

## Abbreviations

The following abbreviations are used in this manuscript:

ABC	ATP-Binding Cassette
ABCA1	Adenosine Triphosphate Binding Cassette A1
ADME	Absorption, Distribution, Metabolism, and Excretion
Akt	Protein Kinase B
ARE	Antioxidant Response Element
ATP	Adenosine Triphosphate
Bax	Bcl-2-Associated X Protein
Bcl-2	B-Cell Lymphoma 2
BEO	Bergamot Essential Oil
BEO-BF	Bergapten-Free BEO
BMT	Bergaptol 5-O-Methyltransferase
C2′H	Cinnamoyl-CoA 2′-Hydroxylase
C4H	Cinnamate 4-Hydroxylase
CAT	Catalase
CDK2	Cyclin-Dependent Kinase 2
CDK4	Cyclin-Dependent Kinase 4
COX-2	Cyclooxygenase-2
CPC	Centrifugal Partition Chromatography
DMAPP	Dimethylallyl Pyrophosphate
DMBA	Dimethylbenz(a)Anthracene
DNA	Deoxyribonucleic Acid
DPPC	Dipalmitoylphosphatidylcholine
EPR	Enhanced Permeability and Retention
ERα	Estrogen Receptor α
FASN	Fatty Acid Synthase
G6PDH	Glucose-6-Phosphate Dehydrogenase
GSK-3β	Glycogen Synthase Kinase 3β
HO-1	Heme Oxygenase-1
HPLC	High-Performance Liquid Chromatography
HSCCC	High-Speed Counter-Current Chromatography
IDOL	Inducible Degrader of LDL Receptor
IL	Interleukin
iNOS	Inducible Nitric Oxide Synthase
IUPAC	International Union of Pure and Applied Chemistry
JAK	Janus Kinase
JAK2	Janus Kinase 2
LC3-I	Microtubule-Associated Protein 1 Light Chain 3
LDH	Lactate Dehydrogenase
LDLR	Low-Density Lipoprotein Receptor
LPS	Lipopolysaccharide
LXR	Liver X Receptors
MAE	Microwave-Assisted Extraction
MAPK	Mitogen-Activated Protein Kinase
MDA	Malondialdehyde
mTOR	Mammalian Target of Rapamycin
NDEA	N-Nitrosodiethylamine
NF-κB	Nuclear Factor Kappa B
NF-Y	Nuclear Factor-Y
NO	Nitric Oxide
NOX4	NADPH Oxidase 4
Nrf2	Nuclear Factor Erythroid 2-Related Factor 2
OXPHOS	Oxidative Phosphorylation
p38MAPK	p38 Mitogen-Activated Protein Kinase
PAL	Phenylalanine Ammonia-Lyase
PARP	Poly(ADP-Ribose) Polymerase
PFK1	6-Phosphofructo 1-Kinase
PGE_2_	Prostaglandin E2
PI3K	Phosphoinositide 3-Kinase
PTEN	Phosphatase Tensin Homologue Deleted from Chromosome 10
PTK2	Protein Tyrosine Kinase 2
PUMA	p53 Upregulated Modulator of Apoptosis
PUVA	Psoralen-Ultraviolet A
ROS	Reactive Oxygen Species
SAM	S-Adenosyl-L-Methionine
SASP	Senescence Associated Secretory Phenotype
SCD1	Stearoyl-CoA Desaturase-1
SLNs	Solid Lipid Nanoparticles
SOD	Superoxide Dismutase
SPE	Solid-Phase Extraction
SREBP	Sterol Regulatory Element Binding Protein
STAT	Signal Transducer and Activator of Transcription
TGF-β	Transforming Growth Factor-β
TLR4	Toll-Like Receptor 4
TNF-α	Tumor Necrosis Factor-α
UVA	Ultraviolet A
UV	Ultraviolet
WSZG	Wenshen Zhuanggu Formula
4CL	4-Coumarate-CoA Ligase
5-MOP	5-Methoxypsoralen
8-MOP	8-Methoxypsoralen
